# TRIM proteins in blood cancers

**DOI:** 10.1007/s12079-017-0423-5

**Published:** 2017-11-06

**Authors:** Lisa J. Crawford, Cliona K. Johnston, Alexandra E. Irvine

**Affiliations:** Centre for Cancer Research and Cell Biology, Queen’s University Belfast, 97 Lisburn Road, Belfast, BT9 7BL UK

**Keywords:** TRIM proteins, Ubiquitin, E3 ligase, Leukaemia, Lymphoma, Multiple myeloma

## Abstract

Post-translational modification of proteins with ubiquitin plays a central role in regulating numerous cellular processes. E3 ligases determine the specificity of ubiquitination by mediating the transfer of ubiquitin to substrate proteins. The family of tripartite motif (TRIM) proteins make up one of the largest subfamilies of E3 ligases. Accumulating evidence suggests that dysregulation of TRIM proteins is associated with a variety of diseases. In this review we focus on the involvement of TRIM proteins in blood cancers.

## Introduction

Ubiquitination is a post-translational modification involving the covalent conjugation of one or more ubiquitin molecules to a substrate protein. The attachment of ubiquitin to a protein is important for the regulation of many cellular processes; it can mark a protein for degradation through the 26S proteasome, modify interactions with other proteins, alter cellular localisation or affect activity. The process of ubiquitination is catalysed by the sequential action of three types of enzymes. An E1 enzyme activates ubiquitin and transfers it to an E2 conjugating enzyme, E2 enzymes then work in conjunction with E3 ubiquitin ligases to transfer ubiquitin to a target protein. It is generally accepted that E3 ligases are responsible for substrate recognition and therefore confer specificity to the system (Yau and Rape 2016). Over 600 E3 ligases have been characterised in humans and are classified into 3 different classes: homologous to E6-AP carboxyl terminus (HECT), really interesting new gene (RING) and RING-between-RING (RBR) (Buetow and Huang 2016). RING proteins are the largest class of E3 ligases and among them the tripartite motif (TRIM) family of proteins represent the largest subfamily of RINGs. TRIM proteins are involved in many biological processes including transcriptional regulation, cell proliferation and differentiation, intracellular signalling, apoptosis and immune signalling. Thus, it is not surprising that alterations of TRIM proteins are associated with a variety of pathologies including developmental disorders, inflammatory diseases and cancers. This review will provide an overview of the TRIM family and discuss alterations in TRIM proteins that are implicated in blood cancers.

## TRIM family overview

**Fig. 1:**
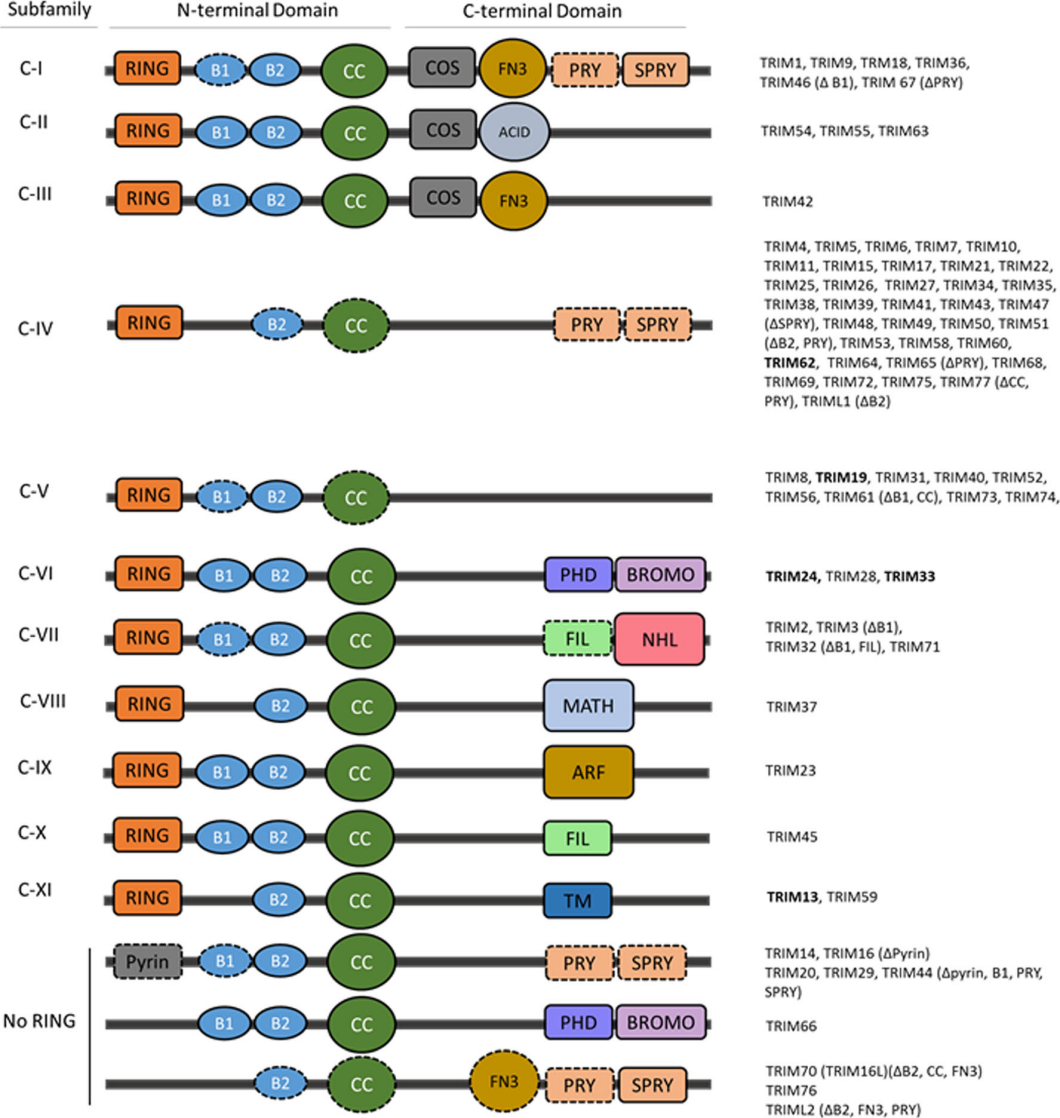
Structural classification of tripartite motif (TRIM) family proteins. The majority of TRIM proteins contain an N-terminal RING domain, one or two B-box domains (B1, B2) and a coiled-coil domain (CC) and are classified into 11 subfamilies (C-I – C-XI) based on a variable C-terminal domain; there is an additional unclassified group lacking a RING domain. Some family members lack one or more domain as denoted in brackets and by a dashed outline. TRIMs included in this review are in bold. Abbreviations: ACID – acid-rich region, ARF – ADP-ribosylation factor family domain, BROMO – bromodomain, COS – cos-box, FIL – filamin-type I G domain, FN3 – fibronectin type III repeat, MATH – meprin and TRAF-homology domain, NHL – NCL1, HT2A and LIN41 domain, PHD - PHD domain, PRY – PRY domain, SPRY – SPRY domain, TM – transmembrane region

TRIM proteins, also referred to as RBBC proteins, are characterised by the presence of an N-terminal tripartite or RBBC motif comprised of a RING domain, either one or two B-boxes (B1 and B2), and a coiled-coil (CC) domain, followed by a highly variable C-terminal domain (Reymond et al. 2001; Torok and Etkin 2001). To date, more than 70 TRIM family members have been identified and these are categorised into 11 subgroups (C-I – C-XI) based on the type of C-terminal domain present (Short and Cox 2006; Ozato et al. 2008); a number of TRIMs lacking a RING domain remain unclassified (Fig. 1). Based on the presence of a RING domain, the majority of TRIMs are defined as E3 ligases (Meroni and Diez-Roux 2005). However it should be noted that the ability to mediate the conjugation of the ubiquitin-like modifications, SUMO and ISG15, is also attributed to a small number of TRIMs (Zou and Zhang 2006; Chu and Yang 2011). The RING domain is a specialised type of zinc finger that confers E3 ligase activity by binding to an ubiquitin-loaded E2 enzyme and promoting the transfer of ubiquitin to a target protein. The B-box domains, in common with the RING domain, are zinc fingers, however, their structural and functional role is less well defined. It has been suggested that due to similarities to the RING domain, they could enhance the E3 ligase activity and even confer E3 ligase activity to RINGless TRIMs, as is the case for TRIM16 (Bell et al. 2012). The coiled-coil domain mediates oligomerisation, that is homo-dimerisation and potentially higher order oligomerisation, a process believed to be required for their function and E3 ligase activity (Esposito et al. 2017). In addition to homo-oligomerisation, there are increasing reports of hetero-oligomerisation between TRIMs, particularly among closely related family members. This is thought to increase the diversity of substrate specificity (Napolitano and Meroni 2012). The variable C-terminal region of TRIM proteins is predominantly responsible for substrate recognition and cellular localisation (Micale et al. 2012). The most prevalent C-terminal is the PRY-SPRY domain found in subfamilies C-I and C-IV, this domain is common in immune signalling proteins (James et al. 2007). Other common C-terminal domains include a COS box found in subfamilies C-I, C-II and C-III and a fibronectin type III (FN3) domain found in subfamilies C-I and C-III.

## TRIM proteins in blood cancers

TRIM proteins function in a broad range of cellular processes and there is accumulating evidence implicating members of the TRIM family in the development and progression of various tumour types. A number of TRIM proteins are linked to the development of blood cancers, through chromosomal translocations or dysregulated expression, acting as either a tumour suppressor or oncogene, depending on the cell type. The involvement of TRIM proteins in the aetiology of blood cancers is summarised below and in Table 1.

**Table 1:** Alterations in TRIM family members in blood cancers

TRIM	Blood cancer	Alteration	Suggested effect	Reference
TRIM13	CLL	Gene Deletion	Tumour suppressor	Kapanadze et al. 2000
	MM	Gene Deletion	Oncogene	Gatt et al. 2013
TRIM19	APL	Translocation:RARα	Tumour suppressor	Chen and Chen 1992
	B-ALL	Translocation:PAX5	Tumour suppressor	Kurahashi et al. 2011
	Lymphoma	Reduced expression	Tumour suppressor	Gurrieri et al. 2004
	CML	Overexpression	Oncogene	Ito et al. 2008
TRIM24	AML	Overexpression	Oncogene	Gandini et al. 2002
	EMS	Translocation:FGFR1	Oncogene	Jackson et al. 2010
TRIM33	CMML	Reduced expression	Tumour suppressor	Aucagne et al. 2011
	B-ALL	N/A	Oncogene	Wang et al. 2015
	MM	Reduced expression	Tumour suppressor	Johnston et al. 2017
TRIM62	AML	Reduced expression	Tumour suppressor	Quintas-Cardama et al. 2015

*AML*, acute myeloid leukaemia; *APL* acute promyelocytic leukaemia; *B-ALL* B cell acute lymphocytic leukaemia; *CLL* chronic lymphocytic leukaemia; *CML* chronic myeloid leukaemia; *CMML* chronic myelomonocytic leukaemia; *EMS* 8p11 myeloproliferative syndrome; *FGFR1* fibroblast growth factor receptor 1; *N/A* not applicable; *PAX5* paired box 5; *RARα* retinoic acid receptor α

## TRIM62

TRIM62 is a member of the largest subfamily of TRIMs, C.IV, which contain a C-terminal PRY/SPRY domain. TRIM62 has been reported to act as a putative tumour suppressor in a number of cancers, including acute myeloid leukaemia (AML). AML is heterogeneous malignancy characterised by the clonal proliferation of immature myeloid cells. It is commonly classified based on cytogenetic and molecular abnormalities (Vardiman et al. 2009). Quintas-Cardama et al. (2015) evaluated TRIM62 protein expression in a large cohort of AML patients at diagnosis. They found that TRIM62 expression was significantly lower in CD34+ cells from AML patients compared to healthy volunteers and low levels were significantly associated with a shorter duration of remission and shorter event-free and overall survival. These effects were particularly notable among patients defined as having cytogenetically normal AML (CN-AML). In this subset of patients a number of molecular aberrations have been found to play an important role in prognosis, this study found that TRIM62 represents an additional independent adverse prognostic factor in CN-AML. While the mode of action of TRIM62 has not been defined, low TRIM62 levels were associated with altered expression of proteins involved in stem cell homeostasis, cell motility and adhesion, hypoxia and apoptosis.

## TRIM19

TRIM19 is more commonly known as the promyelocytic leukaemia (PML) protein as it was originally identified as part of a balanced translocation with retinoic acid receptor α (RARα) that specifically occurs in acute promyelocytic leukaemia (APL) (de The et al. 1991). It belongs to the C-V subfamily of TRIM proteins, which lack any obvious domain other than R-B1-B2-CC domain that is common to all TRIMs. In normal cells, PML is essential for the formation of distinct nuclear structures known as PML nuclear bodies (PML-NB). These are dynamic structures that are triggered in response to various cellular stresses. PML-NBs are implicated in the regulation of a wide range of cellular processes including transcriptional regulation, cell cycle control, apoptosis, senescence, DNA damage response and anti-viral response (Bernardi and Pandolfi 2007). There are at least 7 protein isoforms of PML which all share an identical N-terminal TRIM motif and have varying C-terminals. However, the majority of isoforms contain a nuclear localisation signal and SUMO-interacting motif (SIM) which is critical for PML-NB formation (Li et al. 2017).

### TRIM19 in acute promyelocytic leukaemia

Acute promyelocytic leukaemia (APL) is a distinct subtype of AML characterised by the accumulation of abnormal promyelocytes in the bone marrow. In the majority of patients (>98%), APL is associated with a balanced reciprocal chromosomal translocation, t(15;17), which produces the PML-RARα fusion protein (de The et al. 1991). RARα is a nuclear receptor that regulates transcription in a ligand-dependent manner. When bound to retinoic acid (RA), RARα induces the expression of genes promoting myeloid differentiation and conversely in the absence of RA, RARα represses the transcription of target genes. The PML-RARα fusion protein retains the N-terminal multimerisation domain of PML and the C-terminal DNA and ligand-binding domain of RARα and acts in a dominant negative manner to disrupt the function of both proteins (Chen and Chen 1992). Through the formation of PML-RARα/PML heterodimers, PML-RARα antagonises the formation of PML-NBs. In addition, PML-RARα acts as a transcriptional suppressor of RARα function, thus inducing a block in differentiation of promyelocytes. Historically, APL conferred a poor prognosis, however, the introduction of therapies specifically targeting PML-RARα has dramatically improved outcomes. Two targeted therapies, all-trans retinoic acid (ATRA) and arsenic trioxide (ATO), each act on one partner of the PML-RAR fusion protein (Zhou et al. 2007). ATRA induces dissociation of co-repressor complexes from the RARα moiety and subsequently induces proteasome-mediated degradation of PML-RARα. This promotes differentiation of leukaemic promyelocytes into mature granulocytes. ATO on the other hand binds to PML and PML-RARα resulting in sumoylation which in turn promotes polyubiquitination and degradation of PML-RARα (Tomita et al. 2013). There is a high degree of synergy between these targeted agents and they are commonly incorporated into APL induction therapies (Abaza et al. 2017; Platzbecker et al. 2017).

### TRIM19 in other haematological malignancies

In addition to its role in APL, PML exhibits dysregulated expression in other haematopoietic malignancies. PML has been found as a translocation partner with the transcription factor paired box 5 (PAX5) [t(9;15)] in some cases of B-cell acute lymphocytic leukaemia (B-ALL). Similar to PML-RARα, the PAX-PML fusion protein acts in a dominant negative manner to inhibit PAX5 transcriptional activity and impair the formation of PML-NBs (Kurahashi et al. 2011). Furthermore, reduced protein expression of PML has been reported in non-Hodgkin’s lymphoma (Gurrieri et al. 2004). A role for PML in lymphoma was further demonstrated using mouse models of myc-driven B-lymphoma. Loss of one allele of E6AP, an E3 ligase for PML, restored PML levels and induced cellular senescence, leading to a significant delay in Myc-induced lymphagenesis.

Overall, these studies are in agreement with PML’s widely reported function as a tumour suppressor in a variety of both haematopoietic and solid tumours (Gamell et al. 2014), however, chronic myeloid leukaemia (CML) is an exception to this. CML is a myeloproliferative disorder that, in common with APL, is characterised by a specific chromosomal translocation, in this case t(9;22), which gives rise to the tyrosine kinase BCR-ABL. The translocation originates in haematopoietic stem cells that can self-renew, proliferate and differentiate leading to an excessive accumulation of myeloid cells. Tyrosine kinase inhibitors (TKIs) target the kinase activity of BCR-ABL and have transformed the treatment of CML, however, the persistence of TKI-resistant stem cells remains one of the major obstacles to eradicating the disease (Holyoake and Vetrie 2017). Ito et al. (2008) found that PML was required for the maintenance of both normal haematopoietic stem cells and leukaemic stem cells expressing BCR-ABL. Moreover, they reported that low expression of PML in CML blasts was associated with improved overall survival. A number of laboratory studies point to the potential of using ATO in combination with TKIs to target CML stem cells (Naka et al. 2010) Fig. 2.

**Fig. 2:**
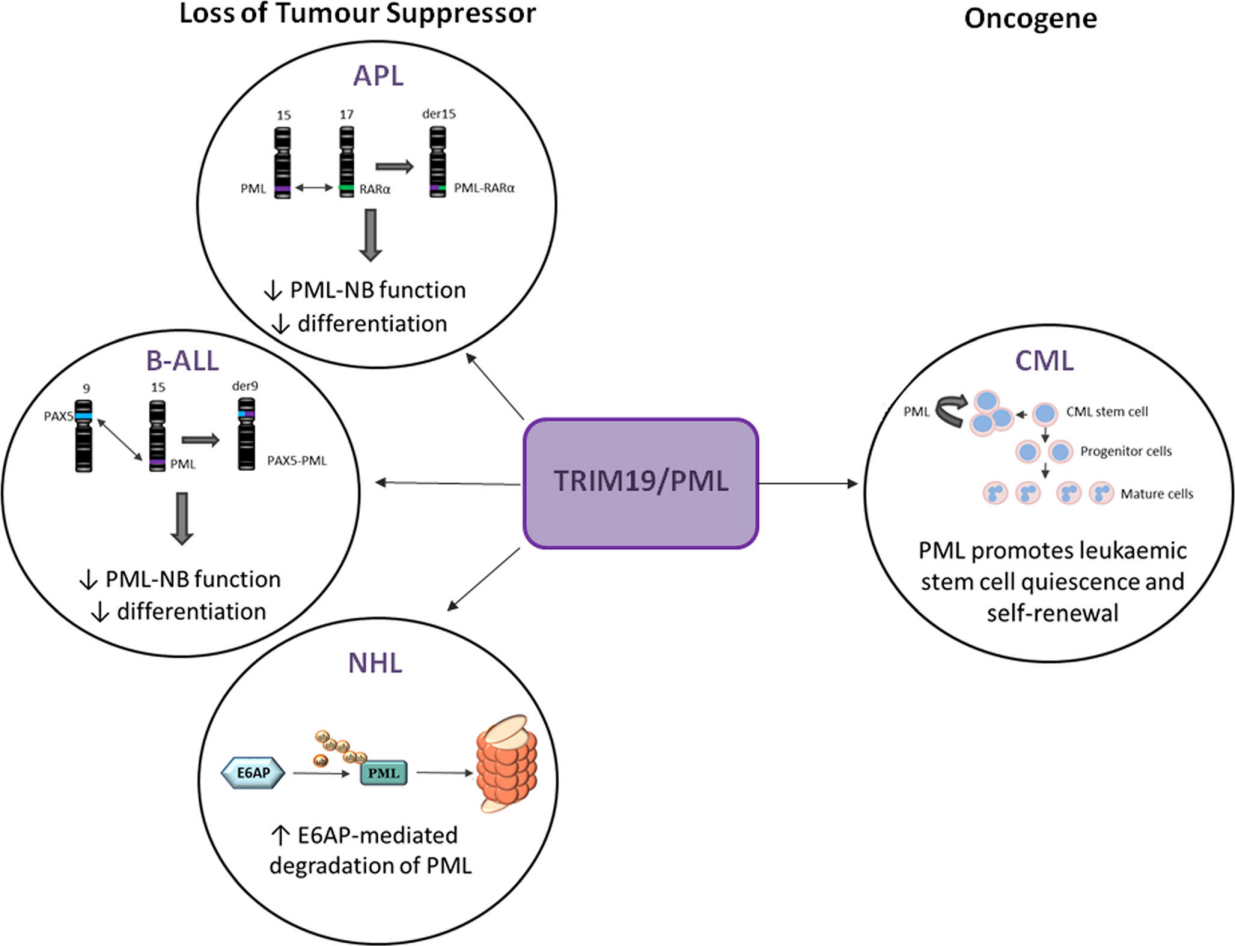
TRIM19/PML exerts cell type dependent effects on blood cancers. In acute promyelocytic leukaemia (APL), B cell acute lymphoblastic leukaemia (B-ALL) and non-Hodgkin’s lymphoma (NHL), the tumour suppressive activity of PML is lost. The majority of patients with APL harbour the t(15;17) translocation resulting in a promyelocytic leukaemia – retinoic acid receptor α (PML-RARα) gene and protein. This disrupts the normal function of both proteins leading to impaired PML-nuclear bodies (PML-NB) formation and a block in differentiation. In some cases of B-ALL, PML is found translocated to the transcription factor paired box 5 (PAX5), again disrupting the formation of PML-NB and leading to decreased differentiation. In NHL reduced protein expression of PML is seen due to increased E6AP-mediated proteasomal degradation of PML. Conversely, PML is seen to play an oncogenic role in chronic myeloid leukaemia (CML), where it has been found to be important for self-renewal and quiescence in leukaemic stem cells

## TRIM33

TRIM33, also known as transcriptional intermediary factor 1 γ (TIF1γ), is a nuclear protein that belongs to the C-VI subfamily of TRIM proteins containing a C-terminal tandem plant homeodomain (PHD) and bromodomain module. Members of this subfamily also belong to the TIF1 family, which are involved in chromatin-mediated transcriptional regulation. The PHD-bromodomain module interacts with post-translationally modified histone tails to recruit TRIM33 to chromatin and stimulate its E3 ligase activity (Agricola et al. 2011). TRIM33 is implicated in the regulation of many aspects of haematopoiesis and interacts with key transcriptional regulators of haematopoiesis, such as PU.1, TAL1 and SMAD4. The importance of TRIM33 in haematopoiesis was first reported in a zebrafish model demonstrating that loss of TRIM33 disrupts both embryonic and adult haematopoiesis and is required for normal erythroid development (Ransom et al. 2004). Subsequent studies have found that TRIM33 is involved in the regulation of haematopoietic stem cells (HSC), granulomonopoiesis and macrophage differentiation, transcriptional elongation of erythroid genes (Bai et al. 2010, 2013) and plays a key role in the recruitment of myeloid cells to sites of inflammation (Demy et al. 2017). It is therefore not surprising that TRIM33 has also been found to be dysregulated in a number of haematological malignancies. While TRIM33 is largely considered to exhibit tumour suppressor activity, it is also reported to act as oncogene depending on the cell type involved.

### TRIM33 in chronic myelomonocytic leukaemia

Chronic myelomonocytic leukaemia (CMML) is a clonal HSC malignancy characterised by the expansion of the granulo-monocytic compartment in the bone marrow, peripheral blood and spleen. CMML is generally associated with advancing age and has a median age at diagnosis of 70 (Padron et al. 2015). Using a conditional knockout model, Aucagne et al. (2011) demonstrated that loss of TRIM33 in mouse HSCs favoured the expansion of granulo-monocytic progenitors and older TRIM33-deficient mice exhibited features of CMML. Furthermore, they demonstrated that TRIM33 was downregulated in 35% of CMML patients. Low levels of TRIM33 in CMML patients were due to hypermethylation of the gene promoter and expression could be restored using the hypomethylating agent decitabine. The hypomethylating agents azacitidine and decitabine are commonly used to treat older patients with CMML (Alfonso et al. 2017) and have recently been shown to confer a significant survival advantage to CMML patients in the first year after diagnosis (Zeidan et al. 2017). Future studies are warranted to investigate whether TRIM33 could be used as a biomarker of response to these agents in CMML.

### TRIM33 in B-cell acute lymphoblastic leukaemia

In contrast to the role of TRIM33 as a tumour suppressor in CMML, Wang et al. (2015) found that TRIM33 acts as an oncogene in B-cell acute lymphoblastic leukaemia (B-ALL), a malignancy of immature B-cells that predominantly affects young children. TRIM33 was identified during an RNAi screen for essential chromatin regulators in B-ALL. In murine B-ALL cells, it was demonstrated that TRIM33 is recruited by PU.1 to select lineage-specific enhancers and inhibits apoptosis in these cells by blocking activation of the pro-apoptotic gene *Bim*. TRIM33-deficient mice displayed selective loss of CD19 and B220 B lymphoid cells suggesting that TRIM33 is also required for normal B cell development. While the authors propose that TRIM33 is essential for the survival of all B cell neoplasms, there is conflicting evidence to suggest that TRIM33 has opposing effects in Multiple Myeloma (MM). MM is characterised by the clonal proliferation of plasma cells, which are terminally differentiated B cells. TRIM33 is located on chromosome 1p13.1, a common deleted region seen in MM patients (Li et al. 2016). Low expression of TRIM33 in MM has previously been found to be significantly associated with poor clinical outcome (Shaughnessy et al. 2007). In concert with this, we have recently proposed that TRIM33 acts as a tumour suppressor in MM (Johnston et al. 2017 and unpublished observations). There are well recognised functional and transcriptional differences that distinguish B cells from more differentiated antibody secreting plasma cells and this may account for the opposing roles of TRIM33 suggested in B-ALL versus MM cells (Recaldin and Fear 2016; Nutt et al. 2015) Fig. 3.

**Fig. 3:**
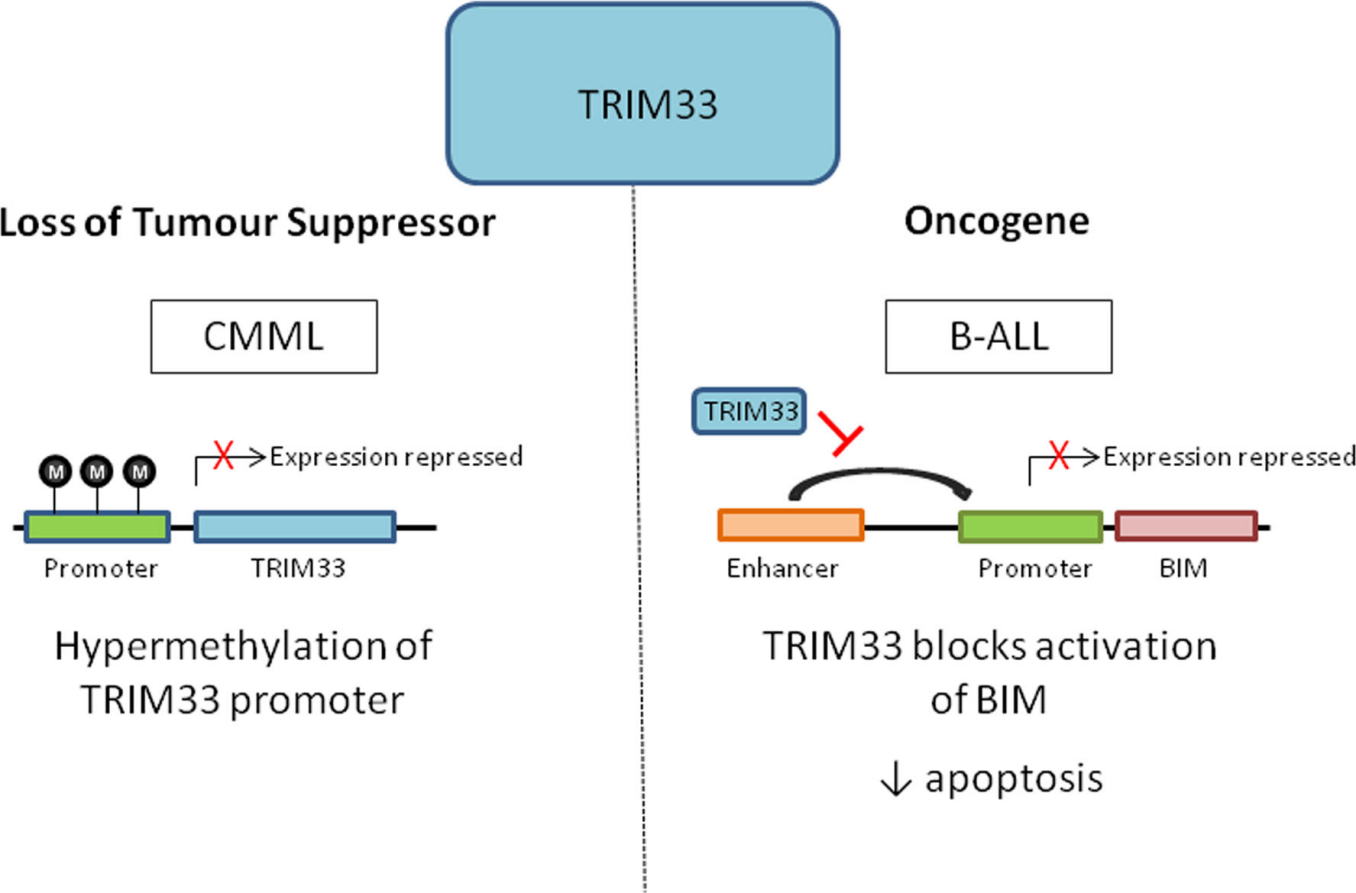
The dual role of TRIM33 in the regulation of blood cancers. In chronic myelomonocytic leukaemia (CMML) TRIM33 is epigenetically silenced through increased methylation of its promoter in approximately a third of patients, leading to loss of tumour suppressor activity. Conversely, TRIM33 has been found to be essential for the survival of B cell acute lymphoblastic leukaemia (B-ALL) cells by blocking enhancer mediated activation of the pro-apoptotic gene *BIM*

## TRIM24

TRIM24, also known as TIF1α, is also a member the C-VI subfamily of TRIM proteins and the TIF1 family. In common with TRIM33, TRIM24 is involved in chromatin-dependent regulation of transcription, however, they differ in their specificity for histone modifications (Herquel et al. 2011a, b). TRIM24 regulates cellular transcription by interacting with nuclear receptors such as retinoic acid and is also involved in controlling the stability of the tumour suppressor p53. Like many other TRIM proteins, TRIM24 has been reported to exhibit both tumour suppressor and oncogenic properties in a cell type dependent context. Due to its interactions with retinoic acid receptors, it has been postulated that TRIM24 may play a role in myeloid differentiation. While there is no direct evidence for this, dysregulated expression of TRIM24 has been reported in AML. Gandini et al. (2002) found higher expression of TRIM24 in some subtypes of AML and they report significantly overexpressed TRIM24 in AML that has transformed from a pre-leukaemic haematological disorder known as myelodysplastic syndrome (MDS) but not in untransformed MDS. TRIM24 has also been implicated in recurrent chromosomal rearrangements associated with a neoplasm known as 8p11 myeloproliferative syndrome or EMS (Belloni et al. 2005). This is a rare myeloproliferative neoplasm associated with translocation of Fibroblast Growth Factor Receptor 1 (FGFR1) on chromosome 8p11 to one of at least 14 partner genes, resulting in constitutive tyrosine kinase activity (Jackson et al. 2010). EMS is characterized by eosinophilia, T-cell proliferation and frequent progression to AML (Patnaik et al. 2010).

## TRIM13

TRIM13, also known as ret finger protein 2 (RFP2), belongs to the C-XI subfamily containing a RING finger, one B-box domain, a coiled-coil domain and a C-terminal transmembrane domain. It localises to endoplasmic reticulum (ER) membranes via its transmembrane domain and regulates the turnover of ER-associated degradation (ERAD) substrates. TRIM13 was initially identified as a putative tumour suppressor gene in B cell chronic lymphocytic leukaemia (B-CLL) (Kapanadze et al. 2000). It is located on chromosome 13 within a minimum commonly deleted region (13q14) that is frequently deleted in CLL and also lost in other B cell malignancies such as MM, mantle cell lymphoma (MCL) and diffuse large B cell lymphoma (DLBCL). While TRIM13 was first identified as a candidate tumour suppressor in B-CLL, there are conflicting reports on its relevance. Following initial identification, a number of studies have excluded TRIM13 as a tumour suppressor gene in B-CLL (Rondeau et al. 1999; Bullrich et al. 2001). However, Baranova et al. (2003) subsequently reported downregulated expression of TRIM13 in CLL patients at advanced stage of disease in comparison to expression at diagnosis, suggesting that it does exhibit properties of a tumour suppressor. In MM, deletion of chromosome 13q, particularly 13q14, is present in approximately half of patients at diagnosis and is associated with a poor clinical outcome. Gatt et al. (2013) investigated the role of TRIM13 in the pathogenesis of MM using a loss-of-function approach. Unexpectedly they found that TRIM13 downregulation led to decreased survival and proliferation of MM cell lines, along with inhibition of the NFκB pathway and proteasome activity.

## Concluding remarks

As described in this review, TRIM proteins can positively or negatively regulate the initiation or progression of blood cancers by affecting processes such as transcriptional regulation, cell cycle control, differentiation and apoptosis. In fact, many of the TRIM proteins described can exhibit a dual role either as an oncogene or tumour suppressor, depending on the context. For example, loss of the normal function of TRIM19/PML through translocation or E6AP-mediated degradation is implicated in the pathogenesis of APL, B-ALL and lymphoma, whereas in CML low TRIM19 expression correlates with improved overall survival. This demonstrates that the role of TRIM proteins in the regulation of both normal haematopoiesis and leukaemogenesis is complex and often cell type specific, highlighting the need to investigate the biological relevance of individual TRIMs in the appropriate cell type. To add a further layer of complexity, a number of TRIM proteins are known to heterodimerise with other TRIMs, which is thought to enhance their E3 ligase activity or diversify substrate specificity. Two TRIMs, TRIM24 and TRIM33, described in isolation in this review are known to form a complex that co-operatively acts in tumour suppression in hepatocellular cancer cells (Herquel et al. 2011b). Investigation of the role of this heterodimer in relevant blood cancers could reveal additional roles in the promotion or prevention of oncogenesis. Over half of TRIM family members, including those discussed here, have been shown to play an important role in regulating the innate immune response. The immune system is known to be involved in shaping the evolution and biology of blood cancers and as our understanding of the role of TRIM proteins in immunity increases, there may be more TRIMs identified that indirectly participate in tumour development and progression. Given their involvement in a variety of diseases, TRIM proteins represent attractive therapeutic targets. The ubiquitin proteasome system is already established as an important therapeutic target in blood cancers. Inhibition of proteasome activity with bortezomib or carfilzomib is widely used in all aspects of anti-MM therapies (Landgren and Iskander 2017). Furthermore, there is increasing interest in targeting individual E3 ligases either through inhibiting or altering E3 ligase activity The successful use of ATO to treat APL represents the first use of a TRIM-specific targeted therapy. As our knowledge of the role of TRIM family members in blood cancers increases, it is likely that further opportunities for the development of targeted therapies will emerge.
